# Matrix discrepancy and the log-rank conjecture

**DOI:** 10.1007/s10107-024-02117-9

**Published:** 2024-07-05

**Authors:** Benny Sudakov, István Tomon

**Affiliations:** 1 ETH Zurich, Zürich, Switzerland; 2 Umeå University, Umeå, Sweden

**Keywords:** Discrepancy, Log-rank conjecture, 68R05, 68Q17

## Abstract

Given an $m\times n$ binary matrix *M* with $|M|=p\cdot mn$ (where |*M*| denotes the number of 1 entries), define the *discrepancy* of *M* as ${{\,\textrm{disc}\,}}(M)=\displaystyle \max \nolimits _{X\subset [m], Y\subset [n]}\big ||M[X\times Y]|-p|X|\cdot |Y|\big |$. Using semidefinite programming and spectral techniques, we prove that if ${{\,\textrm{rank}\,}}(M)\le r$ and $p\le 1/2$, then

$$\begin{aligned}{{\,\textrm{disc}\,}}(M)\ge \Omega (mn)\cdot \min \left\{ p,\frac{p^{1/2}}{\sqrt{r}}\right\} .\end{aligned}$$

We use this result to obtain a modest improvement of Lovett’s best known upper bound on the log-rank conjecture. We prove that any $m\times n$ binary matrix *M* of rank at most *r* contains an $(m\cdot 2^{-O(\sqrt{r})})\times (n\cdot 2^{-O(\sqrt{r})})$ sized all-1 or all-0 submatrix, which implies that the deterministic communication complexity of any Boolean function of rank *r* is at most $O(\sqrt{r})$.

## Introduction

The log-rank conjecture, proposed by Lovász and Saks [12] in 1988, is one of the fundamental open problems in communication complexity. It states that for any Boolean function $f:X\times Y\rightarrow \{-1,1\}$ of rank *r*, its deterministic communication complexity $\text{ CC}^{det}(f)$ is bounded by ${{\,\textrm{polylog}\,}}(r)$. We refer the reader to Kushilevitz and Nisan [7] for exact definitions, or the recent survey of Lee and Shraibman [10] for a detailed overview of the problem. The log-rank conjecture has a number of combinatorial interpretations, which will be the main focus of our paper.

Lovász and Saks [12] observed (see also [13]) that this problem is closely related to bounding the chromatic number of graphs, whose adjacency matrix has bounded rank. Indeed, if *c*(*r*) denotes the maximum of $\text{ CC}^{det}(f)$ among every *f* of rank *r*, then $\chi (G)\le 2^{c(r+1)}$ for every graph *G*, whose adjacency matrix has rank at most *r*. Furthermore, the log-rank conjecture is related to finding monochromatic submatrices in low-rank binary matrices. To this end, let $\alpha (r)>0$ be such that any binary matrix $M\in \{0,1\}^{m\times n}$ of rank at most *r* contains an all-0 or all-1 submatrix of area at least $mn/2^{\alpha (r)}$ (here, the *area* of a matrix refers to the product of the number of rows and columns). It was proved by Nisan and Wigderson [15] that $c(f)=O((\log r)^2+\sum _{i=0}^{\log _2 r}\alpha (r/2^i))$.

The best known lower bound $c(r)=\tilde{\Omega }((\log r)^2)$ is due to Göös, Pitassi, and Watson [4]. On the other hand, the best known upper bound was due to Lovett [13], who showed that $\alpha (r)\le O(\sqrt{r}\cdot \log r)$, and thus $c(r)=O(\sqrt{r}\cdot \log r)$. Here, we provide the modest improvement that $c(r)=O(\sqrt{r})$ by establishing the following improvement on $\alpha (r)$.

### Theorem 1.1

There exists a constant $c>0$ such that the following holds. Let $M\in \{0,1\}^{m\times n}$ such that ${{\,\textrm{rank}\,}}(M)\le r$. Then there exists $X\subset [m]$ and $Y\subset [n]$ such that

$$\begin{aligned}|X|\ge \frac{m}{2^{c\sqrt{r}}} \text{ and } |Y|\ge \frac{n}{2^{c\sqrt{r}}},\end{aligned}$$

and $M[X\times Y]$ contains only 0 or only 1 entries.

One of the key components of the proof of Lovett [13] is a result of Linial, Mendelson, Schechtman, and Shraibman [9] (see also [10]) on the *discrepancy* of low-rank matrices. Given a matrix $M\in \{-1,1\}^{m\times n}$, this discrepancy is defined as

$$\begin{aligned}{{\,\textrm{disc}\,}}^{*}(M)=\min _{\mu }\max _{\begin{array}{c} X\subset [m]\\ Y\subset [n] \end{array}}\left| \sum _{(x,y)\in X\times Y}M_{x,y}\cdot \mu (x,y)\right| ,\end{aligned}$$

where $\mu$ ranges over all distributions on $X\times Y$. In [9], it is proved that ${{\,\textrm{disc}\,}}^{*}(M)\ge \frac{1}{8\sqrt{r}}$ if ${{\,\textrm{rank}\,}}(M)=r$. Starting with a matrix where at most half of the entries are 1, Lovett [13] applies this inequality to pass to sparser and sparser submatrices iteratively (where sparsity measures the number of 1 entries) until we reach a certain sparsity where the problem becomes easy.

Our main novelty is that we study an alternative definition of discrepancy, inspired by the one commonly used in graph theory [2, 16]. This notion of discrepancy is more sensitive to the sparsity of our matrix, which allows us to find significantly sparser submatrices in already sparse matrices. Given a binary matrix $M\in \{0,1\}^{m\times n}$, let |*M*| denote the number of 1 entries of *M*. Writing $p=\frac{|M|}{mn}$, define the *discrepancy* of *M* as

$$\begin{aligned}{{\,\textrm{disc}\,}}(M)=\max _{X\subset [m], Y\subset [n]}\big ||M[X\times Y]|-p|X|\cdot |Y|\big |.\end{aligned}$$

Viewing *M* as the bipartite graph, whose vertex classes are the rows and columns, and whose edges correspond to the 1 entries of *M*, this definition of discrepancy is a bipartite analogue of the graph discrepancy introduced in the 80’s by Erdős, Goldberg, Pach, and Spencer [2] and extensively studied since then. Our main contribution is the following lower bound on the discrepancy of low-rank matrices.

### Theorem 1.2

There exists a constant $c>0$ such that the following holds. Let $M\in \{0,1\}^{m\times n}$ such that ${{\,\textrm{rank}\,}}(M)\le r$. If $p=\frac{|M|}{mn}\le 1/2$, then ${{\,\textrm{disc}\,}}(M)\ge cmn\cdot \min \left\{ p,\frac{p^{1/2}}{\sqrt{r}}\right\}$.

Note that this bound is the best possible. In case $p\gg 1/r$, i.e. when the minimum is attained by $p^{1/2}/\sqrt{r}$, consider a random $r\times r$ binary matrix *R* in which each entry is 1 with probability *p*. It is easy to show that ${{\,\textrm{disc}\,}}(R)=O(\sqrt{p}r^{3/2})$ with high probability, see the Concluding remarks for a sketch proof of this statement. For every *m*, *n* that are divisible by *r*, then one can construct the $m\times n$ matrix *M* by repeating each row of *R* *m*/*r*-times, and repeating each column *n*/*r*-times. Then ${{\,\textrm{rank}\,}}(M)\le r$ and ${{\,\textrm{disc}\,}}(M)=\frac{mn}{r^2}\cdot {{\,\textrm{disc}\,}}(R)=\Theta (mn p^{1/2}/\sqrt{r})$. On the other hand, if $p\ll 1/r$, then the minimum is attained by *p*. Trivially, |*M*| is always an upper bound on the discrepancy, so our theorem is tight in this case as well.

Equipped with Theorem 1.2, the proof of Theorem 1.1 proceeds via a density decrement argument. As we show in Sect. 2, every $m\times n$ binary matrix *M* contains an $(m/2)\times (n/2)$-sized submatrix $M'$ (disregarding divisibility issues) such that $|M'|\le \frac{1}{4}|M|-\Omega ({{\,\textrm{disc}\,}}(M))$, so large discrepancy implies the existence of a significantly sparser submatrix. To prove this, we introduce and work with the notions of positive and negative discrepancy, showing that they have the same order of magnitude up to a constant factor (see the next section for more details). Now let $M_0=M$ be an $m\times n$ binary matrix of rank *r*, let $p_0=p=\frac{|M|}{mn}$, and assume that $p\le 1/2$. Having defined $M_i$ for some $i\ge 0$ with density $p_i<1/2$, we proceed as follows. If $p_i<1/(8r)$, we can apply a simple combinatorial argument to find an all-zero submatrix of $M_i$ with a quarter of the rows and columns, in which case we stop and output this submatrix. This argument appears in Lemma 4.1. In the other case $p_i>1/(8r)$, Theorem 1.2 implies that $M_i$ contains an $(m/2)\times (n/2)$ sized binary submatrix $M_{i+1}$ of density at most $p_i-\Omega (p_i^{1/2}/\sqrt{r})$. Simple calculations show that we reach a submatrix of density at most 1/(8*r*) in at most $O(\sqrt{r})$ steps, so the last matrix has size $\frac{m}{2^{O(\sqrt{r})}}\times \frac{n}{2^{O(\sqrt{r})}}$. See Sect. 4 for the detailed argument.

In order to prove our bound on the discrepancy of low rank matrices, that is Theorem 1.2, we follow the recent framework of Räty, Sudakov, and Tomon [16]. While their results are concerned about the usual graph theoretic notion of discrepancy, working in a bipartite setting simplifies many of their ideas. We consider a semidefinitie relaxation of the discrepancy, and then apply spectral techniques to prove our lower bounds. We remark that this approach is fairly different from (and perhaps more elementary than) that of Linial, Mendelson, Schechtman, and Schraibman [10], which relies on John’s ellipsoid theorem [6].

## Discrepancy

In this section, we present basic notions and results about matrices and discrepancy, and introduce a semidefinite relaxation. Most of this section is fairly standard, see e.g. [1, 9] for similar results. However, to keep our paper accessible to a wide audience, we provide detailed arguments. We omit floors and ceilings whenever they are not crucial.

An $m\times n$ binary matrix *M* naturally corresponds to the bipartite graph, whose two vertex classes are identified with $[m]=\{1,\dots ,m\}$ and [*n*], and there is an edge between *i* and *j* if $M_{i,j}=1$. While we will use the language of binary matrices, this correspondence is good to keep in mind for many arguments.

Given $M\in \{0,1\}^{m\times n}$, let $p(M)=\frac{|M|}{mn}$ be the *density* of *M*, and let $d(M)=\frac{2|M|}{m+n}$ (which is the average degree of the corresponding bipartite graph). In what follows, fix some $M\in \{0,1\}^{m\times n}$ and write $p=p(M)$. Given $X\subset [m]$ and $Y\subset [n]$, $M[X\times Y]$ is the submatrix of *M* induced by the rows *X* and columns *Y*. Define

$$\begin{aligned}{{\,\textrm{disc}\,}}_{M}(X,Y)={{\,\textrm{disc}\,}}(X,Y)=|M[X,Y]|-p|X||Y|.\end{aligned}$$

Note that if *X* and $X'$ are disjoint, then ${{\,\textrm{disc}\,}}(X\cup X',Y)={{\,\textrm{disc}\,}}(X,Y)+{{\,\textrm{disc}\,}}(X',Y)$. Furthermore, define the *positive discrepancy* of *M* as

$$\begin{aligned}{{\,\textrm{disc}\,}}^{+}(M)=\max _{X\subset [m],Y\subset [n]} {{\,\textrm{disc}\,}}(X,Y)\end{aligned}$$

and the *negative discrepancy* as

$$\begin{aligned}{{\,\textrm{disc}\,}}^{-}(M)=\max _{X\subset [m],Y\subset [n]} -{{\,\textrm{disc}\,}}(X,Y).\end{aligned}$$

Then ${{\,\textrm{disc}\,}}(M)=\max \{{{\,\textrm{disc}\,}}^{+}(M),{{\,\textrm{disc}\,}}^{-}(M)\}$. This is also equal to the so called *cut-norm* of the matrix $M-pJ$, where *J* is the $m\times n$ all-1 matrix. Note that as ${{\,\textrm{disc}\,}}(\emptyset ,\emptyset )=0$, we have ${{\,\textrm{disc}\,}}^{+}(G)\ge 0$ and ${{\,\textrm{disc}\,}}^{-}(G)\ge 0$. Next, we show that ${{\,\textrm{disc}\,}}^{-}(M)$ and ${{\,\textrm{disc}\,}}^{+}(M)$ cannot be too different.

### Claim 2.1

$${{\,\textrm{disc}\,}}^{+}(M)=\Theta ({{\,\textrm{disc}\,}}^{-}(M))$$.

### Proof

Let $X\subset [m]$ and $Y\subset [n]$ such that ${{\,\textrm{disc}\,}}^{+}(M)={{\,\textrm{disc}\,}}(X,Y).$ Let $X'=[m]\setminus X$ and $Y'=[n]\setminus Y$. Then, $0={{\,\textrm{disc}\,}}([m],[n])={{\,\textrm{disc}\,}}(X,Y)+{{\,\textrm{disc}\,}}(X',Y)+{{\,\textrm{disc}\,}}(X,Y')+{{\,\textrm{disc}\,}}(X',Y')$. Hence, at least one of the terms is at most $-{{\,\textrm{disc}\,}}(X,Y)/3$, which shows that ${{\,\textrm{disc}\,}}^{-}(M)\ge {{\,\textrm{disc}\,}}^{+}(M)/3$. Similarly, ${{\,\textrm{disc}\,}}^{+}(M)\ge {{\,\textrm{disc}\,}}^{-}(M)/3$. $\square$

Next, we show that it is enough to consider submatrices of *M* that intersect exactly half of the rows and half of the columns to get the discrepancy up to a constant.

### Claim 2.2

There exists $X_0\subset [m]$ and $Y_0\subset [n]$ such that $|X_0|=m/2$ and $|Y_0|=n/2$, and

$$\begin{aligned}{{\,\textrm{disc}\,}}(X_0,Y_0) \le -{{\,\textrm{disc}\,}}^{-}(M)/12.\end{aligned}$$

### Proof

Let $X\subset [m]$ and $Y\subset [n]$ such that $|{{\,\textrm{disc}\,}}(X,Y)|={{\,\textrm{disc}\,}}(M)$. Assume that ${{\,\textrm{disc}\,}}(X,Y)\ge 0$, the other case can be handled similarly.

First, let us assume that $|X|\le m/2$. Let $X_1$ be a random *m*/2 element subset of [*m*] containing *X*. Then

$$\begin{aligned} \mathbb {E}({{\,\textrm{disc}\,}}(X_1,Y))&={{\,\textrm{disc}\,}}(X,Y)+\mathbb {E}({{\,\textrm{disc}\,}}(X_1\setminus X,Y))\\&={{\,\textrm{disc}\,}}(X,Y)+\frac{m/2-|X|}{m-|X|}{{\,\textrm{disc}\,}}([m]\setminus X,Y)\\&\ge {{\,\textrm{disc}\,}}(X,Y)- \frac{m/2-|X|}{m-|X|}{{\,\textrm{disc}\,}}(X,Y)\ge \frac{1}{2}{{\,\textrm{disc}\,}}(X,Y). \end{aligned}$$

Here, the first inequality holds by the maximality of (*X*, *Y*). Therefore, there exists a choice for $X_1$ such that ${{\,\textrm{disc}\,}}(X_1,Y)\ge \frac{1}{2}{{\,\textrm{disc}\,}}(X,Y)$. In case $|X|\ge m/2$, let $X_1$ be a random *m*/2 element subset of *X*. Similarly as before, by considering the expectation, there exists a choice for $X_1$ such that ${{\,\textrm{disc}\,}}(X_1,Y)\ge \frac{1}{2}{{\,\textrm{disc}\,}}(X,Y)$.

Now let $X_2\subset [m]$ and $Y_1\subset [n]$ such that $|X_2|=m/2$, and $|{{\,\textrm{disc}\,}}(X_2,Y_1)|$ is maximal under these conditions. Assume that ${{\,\textrm{disc}\,}}(X_2,Y_1)\ge 0$, the other case can be handled similarly. Then ${{\,\textrm{disc}\,}}(X_2,Y_1)\ge {{\,\textrm{disc}\,}}(X_2,Y)$. If $|Y_1|\le n/2$, let $Y_2$ be a random *n*/2 element subset of [*n*] that contains $Y_1$. As before, by considering the expectation of ${{\,\textrm{disc}\,}}(X_2,Y_2)$, we get that there is a choice for $Y_2$ such that ${{\,\textrm{disc}\,}}(X_2,Y_2)\ge \frac{1}{2}{{\,\textrm{disc}\,}}(X_2,Y_1)\ge \frac{1}{4}{{\,\textrm{disc}\,}}(X,Y)$. If $|Y_1|\ge n/2$, then we can proceed as above by taking $Y_2$ to be a random *m*/2 element subset of $Y_1$.

In conclusion, we get $X_2\subset [m]$ and $Y_2\subset [n]$ such that $|X_2|=m/2$, $|Y_2|=n/2$, and $|{{\,\textrm{disc}\,}}(X_2,Y_2)|\ge \frac{1}{4}|{{\,\textrm{disc}\,}}(X,Y)|$. If ${{\,\textrm{disc}\,}}(X_2,Y_2)\le 0$, we are done, so we may assume that ${{\,\textrm{disc}\,}}(X_2,Y_2)>0$. Let $X_3=[m]\setminus X_2$, and $Y_3=[n]\setminus Y_2$. Writing the equality $0={{\,\textrm{disc}\,}}(X_2,Y_2)+{{\,\textrm{disc}\,}}(X_3,Y_2)+{{\,\textrm{disc}\,}}(X_2,Y_3)+{{\,\textrm{disc}\,}}(X_3,Y_3)$, at least one of the terms is at most $-{{\,\textrm{disc}\,}}(X_2,Y_2)/3\le -|{{\,\textrm{disc}\,}}(X,Y)|/12$. Hence, we can choose $X_0$ and $Y_0$ such that $|X_0|=n/2$, $|Y_0|=m/2$ and ${{\,\textrm{disc}\,}}(X_0,Y_0)\le -{{\,\textrm{disc}\,}}(M)/12\le -{{\,\textrm{disc}\,}}^{-}(M)/12$. $\square$

Consider the following relaxation of the positive discrepancy. Given $\textbf{x}\in \mathbb {R}^m$ and $\textbf{y}\in \mathbb {R}^n$, let

$$\begin{aligned}{{\,\textrm{disc}\,}}(\textbf{x},\textbf{y})=\textbf{x}^T(M-pJ)\textbf{y}.\end{aligned}$$

Also, define

$$\begin{aligned}{{\,\textrm{disc}\,}}_0(M)=\max _{\textbf{x}\in [-1,1]^m,\textbf{y}\in [-1,1]^n} {{\,\textrm{disc}\,}}(\textbf{x},\textbf{y}).\end{aligned}$$

For a reader familiar with norms, we highlight that ${{\,\textrm{disc}\,}}_0(M)=||M-pJ||_{\infty \rightarrow 1}$.

### Claim 2.3

$${{\,\textrm{disc}\,}}^{+}(M)=\Theta ({{\,\textrm{disc}\,}}_0(M)).$$

### Proof

Let $\textbf{x}\in [-1,1]^m,\textbf{y}\in [-1,1]^n$ be such that ${{\,\textrm{disc}\,}}_0(M)={{\,\textrm{disc}\,}}(\textbf{x},\textbf{y})$. As ${{\,\textrm{disc}\,}}(\textbf{x},\textbf{y})$ is a linear function in every variable, we can find $\textbf{x}\in \{-1,1\}^m$ and $\textbf{y}\in \{-1,1\}^n$ achieving the maximum. Let $X_1=\{i\in [m]:\textbf{x}(i)=1\}$, $X_2=[m]{\setminus } X_1$, $Y_1=\{i\in [n]:\textbf{y}(i)=1\}$ and $Y_2=[n]{\setminus } Y_1$. Then

$$\begin{aligned}{{\,\textrm{disc}\,}}(\textbf{x},\textbf{y})={{\,\textrm{disc}\,}}(X_1,Y_1)-{{\,\textrm{disc}\,}}(X_1,Y_2)-{{\,\textrm{disc}\,}}(X_2,Y_1)+{{\,\textrm{disc}\,}}(X_2,Y_2).\end{aligned}$$

Hence, at least one of the four terms has absolute value at least $\frac{1}{4}{{\,\textrm{disc}\,}}_0(M)$. But this shows that either ${{\,\textrm{disc}\,}}^{+}(M)$ or ${{\,\textrm{disc}\,}}^{-}(M)$ is at least $\frac{1}{4}{{\,\textrm{disc}\,}}_0(M)$, and we are done by Claim 2.1. $\square$

Let us further relax the definition of discrepancy by assigning vector values to the rows and columns. That is, let

$$\begin{aligned}{{\,\textrm{pdisc}\,}}_0(M)=\max \sum _{i,j} M_{i,j}\langle v_i,w_j\rangle -p\sum _{i,j}\langle v_i,w_j\rangle ,\end{aligned}$$

where the maximum is taken over all $v_1,\dots ,v_m,w_1,\dots , w_{n}\in \mathbb {R}^{m+n}$ such that $||v_i||_2,||w_j||_2\le 1$. We note that ${{\,\textrm{pdisc}\,}}_0(M)=\gamma _2^{*}(M-pJ)$, where the interested reader may find more about the $\gamma _2$ and $\gamma _2^*$ norms in [9]. Clearly, ${{\,\textrm{pdisc}\,}}_0(M)\ge {{\,\textrm{disc}\,}}_0(M)$. However, it follows from Grothendieck’s inequality [5] that ${{\,\textrm{pdisc}\,}}_0(M)=O({{\,\textrm{disc}\,}}^{+}(M))$ also holds.

### Lemma 2.4

(Grothendieck’s inequality) There exists a universal constant $K>0$ such that the following holds. For a matrix $Q\in \mathbb {R}^{n\times m}$ let $\beta =\sup \left\{ \sum _{i,j} Q_{i,j}x_iy_j: x_i,\right. \left. y_j \in [-1,1]\right\}$ and $\beta ^{*}=\sup \left\{ \sum _{i,j} Q_{i,j}\langle v_i,w_j\rangle : v_i, w_j,\in \mathbb {R}^{n+m},||v_i||_2\right. \left. \le 1, ||w_i||_2\le 1\right\}$. Then $\beta \le \beta ^{*}\le K\beta$.

### Claim 2.5

$${{\,\textrm{pdisc}\,}}_0(M)=\Theta ({{\,\textrm{disc}\,}}^{+}(M))=\Theta ({{\,\textrm{disc}\,}}^{-}(M))$$

### Proof

Apply Groethendieck’s inequality with the matrix $Q\in \mathbb {R}^{m\times n}$ defined as $Q_{i,j}=M_{i,j}-p$. $\square$

Let $N=m+n$. Let $A\in \mathbb {R}^{N\times N}$ be the adjacency matrix of the bipartite graph corresponding to *M*, and let *L* be the adjacency matrix of the complete bipartite graph on the same vertex set. Formally, *A* is the symmetric matrix defined as $A_{i,j+m}=A_{j+m,i}=M_{i,j}$ for $(i,j)\in [m]\times [n]$, and $A_{i,j}=0$ for $(i,j)\in [m]\times [m]$ and $(i,j)\in [m+1,N]\times [m+1,N]$. Note that ${{\,\textrm{rank}\,}}(A)=2{{\,\textrm{rank}\,}}(M)$. On the other hand, we can write $L=\frac{1}{2}(e\cdot e^{T}-f\cdot f^{T})$, where *e* is the all 1 vector, and $f\in \mathbb {R}^N$ is defined $f(i)=1$ if $i\in [m]$ and $f(i)=-1$ if $i\in [m+1,N]$. Call *A* the *symmetrization* of *M*.

Given a matrix $X\in \mathbb {R}^{N\times N}$, define its discrepancy as

$$\begin{aligned}{{\,\textrm{disc}\,}}_M(X)=\langle X,A\rangle -p\langle X,L\rangle ,\end{aligned}$$

where $\langle .,.\rangle$ denotes the Frobenius (i.e. entry-wise) inner product. Furthermore, define

$$\begin{aligned}{{\,\textrm{pdisc}\,}}(M)=\max \left\{ {{\,\textrm{disc}\,}}_M(X): X \text{ is } \text{ symmetric, } \text{ positive } \text{ semidefinite, } \forall i\in [N], X_{i,i}\le 1\right\} .\end{aligned}$$

Then ${{\,\textrm{pdisc}\,}}(M)=2{{\,\textrm{pdisc}\,}}_{0}(M)$. Indeed, for every *X* satisfying the conditions in the definition of ${{\,\textrm{pdisc}\,}}(M)$, there exist vectors $x_1,\dots ,x_{N}$ of length at most 1 such that $X_{i,j}=\langle x_i,x_j\rangle$, i.e. *X* is the so called *Gram-matrix* of $x_1,\dots ,x_{N}$. But then setting $v_i=x_i$ if $i\le m$, and $w_i=x_{i-m}$ if $m<i\le N$, we have ${{\,\textrm{disc}\,}}_M(X)=2(\sum _{i,j}M_{i,j}\langle v_i,w_j\rangle -p\sum _{i,j}\langle v_i,w_j\rangle ).$ In conclusion, we arrive to the following.

### Claim 2.6

$${{\,\textrm{pdisc}\,}}(M)=\Theta ({{\,\textrm{disc}\,}}^{+}(M))=\Theta ({{\,\textrm{disc}\,}}^{-}(M)).$$

Finally, let us argue that considering only those matrices with equal number of rows and columns does not restrict us too much. Let $M^{\otimes }$ be the $(mn)\times (mn)$ sized matrix we get by repeating each row of *M* *n*-times, and then repeating every column *m* times. It is easy to see that

$$\begin{aligned}\frac{1}{(mn)^2}{{\,\textrm{disc}\,}}_0(M^{\otimes })=\frac{1}{mn}{{\,\textrm{disc}\,}}_0(M),\end{aligned}$$

and $${{\,\textrm{rank}\,}}(M)={{\,\textrm{rank}\,}}(M^{\otimes })$$.

## Low rank matrices

Now let us turn to bounding ${{\,\textrm{pdisc}\,}}(M)$. Let *A* be the adjacency matrix of the bipartite graph corresponding to *M*, defined above. Let $\lambda _1\ge \dots \ge \lambda _{N}$ be the eigenvalues of *A*, and let $v_1,\dots ,v_{N}$ be a corresponding orthonormal set of eigenvectors. Note that as *A* is the adjacency matrix of a bipartite graph, we have $\lambda _i=-\lambda _{N+1-i}$ for $i\in [N]$, and the vectors $v_i(j)$ and $v_{N+1-i}(j)$ agree on [*m*], and are opposite on $[m+1,N]$, see Chapter 11 of [11]. Formally, $v_i(j)=f(j)\cdot v_{N+1-i}(j)$ for every $i,j\in [N]$, where $f(j)=1$ if $j \in [m]$ and $-1$ if $j \in [m+1, N]$. Note that $\lambda _1\ge \dots \ge \lambda _n\ge 0\ge \lambda _{n+1}\ge \dots \ge \lambda _{N}$.

### Lemma 3.1

Let $a_1,\dots ,a_{N}$ be real numbers and let $X=\sum _{i=1}^{N}a_i\cdot v_i\cdot v_i^{T}$. Then

$$\begin{aligned}{{\,\textrm{disc}\,}}_M(X)\ge \sum _{i=1}^{N}a_i\lambda _i-\frac{pN}{2}\cdot \max _{i}(a_i-a_{N+1-i}).\end{aligned}$$

### Proof

Write $A=\sum _{i=1}^{N}\lambda _i v_i\cdot v_i^{T}$, and observe the identity $\langle v\cdot v^{T},w\cdot w^{T}\rangle =\langle v,w\rangle ^2$. Then

$$\begin{aligned}\langle X,A\rangle =\sum _{i=1}^{N}\sum _{j=1}^{N}a_i\lambda _j \langle v_i,v_j\rangle ^2=\sum _{i=1}^{N}\lambda _ia_i.\end{aligned}$$

One the other hand,

$$\begin{aligned}\langle X,L\rangle= & {} \frac{1}{2}\sum _{i=1}^{N}a_i(\langle v_i,e\rangle ^2-\langle v_i,f\rangle ^2)\\= & {} \frac{1}{2}\sum _{i=1}^{N}(a_i-a_{N+1-i})\langle v_i,e\rangle ^2\le \frac{N}{2}\max _i |a_i-a_{N+1-i}|.\end{aligned}$$

Here, the second equality holds by noting that $\langle v_i,e\rangle =\langle v_{N+1-i},f\rangle$, and the last inequality as $\sum _{i=1}^{N}\langle v_i,e\rangle ^2=||e||_2^2=N.$ $\square$

In what follows, let us assume that $m=n$, so $N=2n$. Let $d=d(M)=\frac{|M|}{n}=\frac{pN}{2}$, which is the average degree of the corresponding bipartite graph *G*, and let $\Delta =\Delta (M)$ denote the maximum number of 1 entries in a row or a column, that is, the maximum degree of *G*.

### Lemma 3.2

$${{\,\textrm{pdisc}\,}}(M)\ge \frac{1}{\Delta }\sum _{i=2}^{n} \lambda _i^3.$$

### Proof

Let $X=\frac{1}{\Delta }\sum _{i=1}^{n}\lambda _i^2\cdot v_i\cdot v_i^{T}$, which is positive semidefinite. We also claim that $X_{i,i}\le 1$ for every $i\in [N]$. Indeed, note that $\frac{1}{\Delta }A^2-X=\frac{1}{\Delta }\sum _{i=n+1}^{N}\lambda _i^2\cdot v_i\cdot v_i^{T}$ is positive semidefinite. Therefore all diagonal entries of $\frac{1}{\Delta }A^2-X$ are non-negative. Moreover, the *i*-th diagonal entry of $A^2$ counts the number of 1 in the *i*-th row of *A*. Therefore every diagonal entry of $\frac{1}{\Delta }A^2$ is at most 1. Thus, $X_{i,i}\le 1$ for every $i\in [N]$, which implies that ${{\,\textrm{pdisc}\,}}(M)\ge {{\,\textrm{disc}\,}}_M(X)$. Then, by Lemma 3.1 (with $a_i=\lambda _i^2$ for $1 \le i \le n$ and $a_i=0$ for $n+1 \le i \le N$), we have

$$\begin{aligned} {{\,\textrm{pdisc}\,}}(M)\ge {{\,\textrm{disc}\,}}_M(X)\ge & {} \frac{1}{\Delta }\left( \sum _{i=1}^{n}\lambda _i^3-\frac{pN}{2} \cdot \lambda _1^2\right) = \frac{1}{\Delta }\left( \sum _{i=1}^{n}\lambda _i^3-d\lambda _1^2\right) \\\ge & {} \frac{1}{\Delta }\sum _{i=2}^{n}\lambda _i^3.\end{aligned}$$

In the last inequality, we used the well known result that the maximal eigenvalue $\lambda _1$ of a graph *G* is always at least its average degree *d*, see e.g. [11]. $\square$

With the help of this lemma, we prove that low rank matrices have large discrepancy. Unfortunately, the bound in Lemma 3.2 depends on the maximum degree of the corresponding bipartite graph *G* rather than the average degree, which leads to additional difficulties. However, *G* having many vertices with degree larger than the average, or more generally, *G* being far from regular already implies large discrepancy. Dealing with such matrices requires a different argument, so first we assume that the maximum degree is not too large.

### Lemma 3.3

Let ${{\,\textrm{rank}\,}}(M)\le r$, $d\le n/2$ and $\Delta \le 1.1 d$. Then ${{\,\textrm{pdisc}\,}}(M)\ge \frac{d^{1/2}n^{3/2}}{7\sqrt{r}}$.

### Proof

Note that $\sum _{i=1}^{2n}\lambda _i^2=\text{ tr }(A^2)=2dn$, so $\sum _{i=2}^{n}\lambda _i^2\ge dn-\Delta ^2\ge dn -1.21d^2\ge dn/3$. Here, we used that the maximum degree $\Delta$ of a graph is always at least the largest eigenvalue $\lambda _1$, see e.g. [11]. Moreover, as ${{\,\textrm{rank}\,}}(M)\le r$, we have $\lambda _{r+1}=\dots =\lambda _n=0$, so $\sum _{i=2}^{r}\lambda _i^2\ge dn/3$. Applying the inequality between the quadratic and cubic mean, we get

$$\begin{aligned}\left( \frac{\sum _{i=2}^{r}\lambda _i^3}{r-1}\right) ^{1/3}\ge \left( \frac{\sum _{i=2}^{r}\lambda _i^2}{r-1}\right) ^{1/2}\ge \frac{d^{1/2}n^{1/2}}{\sqrt{3(r-1)}}.\end{aligned}$$

Hence, applying Lemma 3.2, ${{\,\textrm{pdisc}\,}}(M)\ge \frac{1}{1.1 d}\sum _{i=2}^{r}\lambda _i^3\ge \frac{d^{1/2}n^{3/2}}{7\sqrt{r}}$. $\square$

Next, we show how to deal with matrices of large maximum degree. That is, we generalize the previous result by showing that the maximum degree condition can be omitted by slightly changing the lower bound.

### Lemma 3.4

Let ${{\,\textrm{rank}\,}}(M)\le r$, $d\le n/2$. Then ${{\,\textrm{pdisc}\,}}(M)\ge c\cdot \min \left\{ dn,\frac{d^{1/2}n^{3/2}}{\sqrt{r}}\right\}$ for some $c>0$.

### Proof

Let $D=\min \left\{ dn,\frac{d^{1/2}n^{3/2}}{7\sqrt{r}}\right\}$ and $\delta =0.01$. For $i\in [n]$, write $\deg _r(i)$ for the number of 1 entries in row *i*, and let $\deg _c(i)$ be the number of 1 entries in column *i* of *M*. Let $U_r=\{i\in [n]: \deg _r(i)\ge (1+\delta )d\}$, and similarly, let $U_c=\{i\in [n]: \deg _c(i)\ge (1+\delta )d\}$. Finally, write $t_r$ for the number of 1 entries in $M[U_r\times [n]]$, and define $t_c$ analogously. We have

$$\begin{aligned}{{\,\textrm{disc}\,}}(U_r,[n])=t_r-p|U_r|n=\sum _{i\in U_r} (\deg _r(i)-d)\ge \sum _{i\in U_r} \frac{\delta }{2}\cdot \deg _r(i)=\frac{\delta }{2} t_r.\end{aligned}$$

Here, in the inequality, we use that $\deg _r(i)(1-\frac{\delta }{2})\ge \frac{\deg _r(i)}{1+\delta }\ge d$. We can show similarly that ${{\,\textrm{disc}\,}}([n],U_c)\ge \frac{\delta }{2}t_c$. Therefore, if $t_r+t_c\ge 0.01D$, then ${{\,\textrm{disc}\,}}^{+}(M)\ge \frac{\delta }{2}\cdot \max \{t_r,t_c\} \ge \frac{1}{4} 10^{-4} D$. Hence, as ${{\,\textrm{pdisc}\,}}(M)=\Theta ({{\,\textrm{disc}\,}}^{+}(M))$, we may assume that $t_r+t_c< 0.01D$.

Change all 1 entries of *M* that are contained in either $U_r\times [n]$ or $[n]\times U_c$ into a 0 entry, and let $M'$ be the resulting matrix. Then

$$\begin{aligned}|M'|\ge |M|-t_r-t_c\ge |M|-0.01D\ge |M|-0.01dn,\end{aligned}$$

so $d'=d(M')\ge 0.99d$ and $d'\le d$. Furthermore, $\Delta (M')\le (1+\delta )d\le 1.1d'$, and ${{\,\textrm{rank}\,}}(M')\le r$. Therefore, we can apply Lemma 3.3 to conclude that ${{\,\textrm{pdisc}\,}}(M')\ge \frac{d'^{1/2}n^{3/2}}{7\sqrt{r}}\ge D/2$. Let *X* be matrix such that ${{\,\textrm{disc}\,}}_{M'}(X)={{\,\textrm{pdisc}\,}}(M')$, and let $A'$ be the symmetrization of $M'$. Then

$$\begin{aligned} |{{\,\textrm{disc}\,}}_{M}(X)-{{\,\textrm{disc}\,}}_{M'}(X)|&=|\langle X,A-A'\rangle -\frac{d-d'}{n}\langle X,L\rangle |\\&\le \langle J,A-A'\rangle + 2(d-d')n\le 4(|M|-|M'|)\le 0.04D. \end{aligned}$$

Here, the inequality $|\langle X,A-A'\rangle |\le \langle J,A-A'\rangle$ holds by noting that $A-A'$ has only 0 and 1 entries. Hence, ${{\,\textrm{disc}\,}}_{M}(X)\ge {{\,\textrm{disc}\,}}_{M'}(X)-0.04D\ge D/2-0.04D\ge D/4$, finishing the proof. $\square$

Note that the previous lemma immediately implies Theorem 1.2 by considering $M^{\otimes }$ instead of *M*, and using that ${{\,\textrm{pdisc}\,}}(M)=\Theta ({{\,\textrm{disc}\,}}(M))$. It will be convenient to rewrite Lemma 3.4 into a slightly more convenient form.

### Theorem 3.5

There exists $c>0$ such that the following holds. Assume that ${{\,\textrm{rank}\,}}(M)\le r$ and $1/(8r)\le p\le 1/2$. Then there exists $X,Y\subset [n]$ such that $|X|=|Y|=n/2$, and

$$\begin{aligned}p(M[X\times Y])\le p-c\frac{p^{1/2}}{\sqrt{r}}.\end{aligned}$$

### Proof

Note that $p=d/n$, so for $1/(8r)\le p$, we have $\min \{dn,\frac{d^{1/2}n^{3/2}}{\sqrt{r}}\}=\Omega (p^{1/2}n^2/\sqrt{r})$. Therefore, ${{\,\textrm{disc}\,}}^{-}(M)=\Omega ({{\,\textrm{pdisc}\,}}(M))=\Omega (p^{1/2}n^2/\sqrt{r})$ by Lemma 3.4. By Claim 2.2, there exist $X\subset [n]$ and $Y\subset [n]$ such that $|X|=|Y|=n/2$ and $-{{\,\textrm{disc}\,}}(X,Y)=\Omega ({{\,\textrm{disc}\,}}^{-}(M))$. But then if $M'=M[X\times Y]$, we have

$$\begin{aligned}|M'|=p|X||Y|+{{\,\textrm{disc}\,}}(X,Y)\le p|X||Y|-\Omega (p^{1/2}n^2/\sqrt{r}),\end{aligned}$$

which implies

$$\begin{aligned}p(M')=\frac{|M'|}{|X||Y|}\le p-\Omega \left( \frac{p^{1/2}}{\sqrt{r}}\right) .\end{aligned}$$

$$\square $$

## Log-rank conjecture

Let *z*(*M*) denote the largest *z* for which there exist $X\subset [n]$, $Y\subset [n]$, such that $|X|=|Y|=z$ and $M[X\times Y]$ has only 0 entries. Based on a lemma of Gavinsky and Lovett [3], we show that sufficiently sparse low-rank matrices *M* satisfy $z(M)=\Omega (n)$.

### Lemma 4.1

Let $M\in \{0,1\}^{n\times n}$ such that ${{\,\textrm{rank}\,}}(M)\le r$ and $p(M)\le \frac{1}{8r}$. Then $z(M)\ge n/4$.

### Proof

Let $X,Y\subset [n]$ be the set of rows and columns containing more than *n*/(4*r*) entries equal to 1, respectively. As $p(M)\le \frac{1}{8r}$, we have $|X|,|Y|\le n/2$. Let $X'=[n]{\setminus } X$ and $Y'=[n]\setminus Y$. Assume that $z(M[X'\times Y'])< n/4$, then we find a permutation matrix of size $r+1$ in $M[X'\times Y']$, contradicting that ${{\,\textrm{rank}\,}}(M)\le r$.

For $k=1,\dots ,r+1$, we find a $k\times k$ sized permutation matrix greedily. Suppose that we have already found $A\subset X'$ and $B\subset Y'$ such that $|A|=|B|=k$, and $M[A\times B]$ is a permutation matrix. Let $X_0\subset X'$ be the set of all rows that intersect a column in *B* in a 1 entry, and let $Y_0\subset Y'$ be the set of all columns that intersect a row in *A* in a 1 entry. Then $|X_0|,|Y_0|\le kn/(4r)\le n/4$, so $|X'{\setminus } X_0|\ge n/4$ and $|Y'{\setminus } Y_0|\ge n/4$. Since we assumed that $z(M[X'\times Y'])< n/4$, there exists $i\in X'\setminus X_0$ and $j\in Y'\setminus Y_0$ such that $M_{i,j}=1$, which means that $(A\cup \{i\})\times (B\cup \{j\})$ induces a $(k+1)\times (k+1)$ sized permutation matrix in *M*. $\square$

### Theorem 4.2

There exists a constant $c>0$ such that the following holds. Let $M\in \{0,1\}^{n\times n}$ such that ${{\,\textrm{rank}\,}}(M)\le r$ and $p(M)\le 1/2$. Then $z(M)\ge n/2^{c\sqrt{r}}$.

### Proof

We will proceed by a density decrement argument. Let $n_0=n$ and $M_0=M$, and let $c_0$ be the constant given by Theorem 3.5. We define a sequence $M_0,M_1,\dots$ of submatrices of *M* with decreasing density. If $M_{i}\in \{0,1\}^{n_i\times n_i}$ is already defined for $i\ge 0$ with $p_i:=p(M_i)\le p(M)$, and we have $p_i\ge 1/(8r)$, we define $M_{i+1}$ by setting $M_{i+1}=M_i[X\cup Y]$, where $X,Y\subset [n_i]$, $|X|=|Y|=n_i/2$, and

$$\begin{aligned}p(M_i[X\times Y])\le p_i-c_0\frac{p_i^{1/2}}{\sqrt{r}}.\end{aligned}$$

Such *X* and *Y* exist by Theorem 3.5. On the other hand, if $p(M_i)<1/(8r)$, then set $I=i$ and stop.

Observe that $n_i=n/2^i$ and $p_{i+1}\le p_i-c_0\frac{p_i^{1/2}}{\sqrt{r}}$ for $i=0,\dots ,I-1$. This implies that if *C* is a sufficiently large constant, then for every $x\in [1/(8r),1/2]$, the number of indices *i* such that $x\le p_i\le 2x$ is at most $C\sqrt{r}x^{1/2}$. Applying this for every $x=2^{-k}$, we get that

$$\begin{aligned}I\le C\sqrt{r}\sum _{i=1}^{\infty }2^{-i/2}\le 10C\sqrt{r}.\end{aligned}$$

As $p(M_I)\le 1/(8r)$ and $n_I\ge n/2^{I}\ge n/2^{10C\sqrt{r}}$, we have $z(M_I)\ge n_{I}/4=n/2^{c\sqrt{r}}$ by Lemma 4.1 with a suitable constant $c>0$, finishing the proof. $\square$

From this, the proof of Theorem 1.1 is immediate.

### Proof of Theorem 1.1

Let $M\in \{0,1\}^{m\times n}$ such that ${{\,\textrm{rank}\,}}(M)\le r$. Without loss of generality, we may assume that $p(M)\le 1/2$, otherwise we can consider $J-M$. Note that ${{\,\textrm{rank}\,}}(J-M)\le r+1$. Writing $n'=mn$, $M^{\otimes }\in \{0,1\}^{n'\times n'}$ satisfies that $p(M^{\otimes })\le 1/2$. Hence, by the previous theorem, $z(M^{\otimes })\ge n'/2^{c\sqrt{r}}$ for some constant $c>0$. Note that each row of *M* is repeated *n* times in $M^{\otimes }$, and each column is at most *m* times, so we can find $X\subset [m]$ and $Y\subset [n]$ such that $|X|\ge z(M^{\otimes })/n$, $|Y|\ge z(M^{\otimes })/m$, and $M[X\times Y]$ has only 0 entries. But then $|X|\ge m/2^{c\sqrt{r}}$ and $|Y|\ge n/2^{c\sqrt{r}}$, finishing the proof. $\square$

## Concluding remarks

As we promised in the introduction, we sketch a proof that if $p\ge 1/r$, then the random $r\times r$ matrix, in which each entry is 1 with probability *p*, has discrepancy $O(\sqrt{p} r^{3/2})$ with high probability. We use the Multiplicative Chernoff’s bound, which shows that if the random variable *Z* is the sum of independent indicator random variables and $\mu =\mathbb {E}(Z)$, then for every $t\ge 0$, we have

$$\begin{aligned}\mathbb {P}(|Z-\mu |\ge t)\le {\left\{ \begin{array}{ll}2e^{-t^2/(3\mu )} &{} \text{ if } t\le \mu ,\\ 2e^{-t/3} &{} \text{ if } t>\mu .\end{array}\right. }\end{aligned}$$

Using the previous inequality, we get that the density of *M*, $p_0=p(M)$, satisfies $|p_0-p|\le 4\sqrt{\frac{p}{r}}$ with probability at least $1-e^{-r}$. Moreover, for any $X,Y\subset [r]$,

$$\begin{aligned}\mathbb {P}\left( \big ||M[X\times Y]|-p|X||Y|\big |\ge 6\sqrt{p}r^{3/2}\right) \le e^{-2r},\end{aligned}$$

by noting that $6\sqrt{p}r^{3/2}\ge r$. Hence, by the union bound, as there are $4^r$ choices for (*X*, *Y*), we get that with high probability, $|M[X\times Y]|-p|X||Y||\le 6\sqrt{p}r^{3/2}$ holds for every *X* and *Y* simultaneously. But then

$$\begin{aligned}{{\,\textrm{disc}\,}}(M)=\max _{X,Y\subset [r]}|M[X\times Y]|-p_0|X||Y||\le 10\sqrt{p}r^{3/2}\end{aligned}$$

also holds with high probability.

Finally, let us mention the following interesting conjecture from [13], which states that a relaxation of Theorem 4.2 should also hold for matrices that are not necessarily binary. We believe the methods presented in our paper could be useful in making progress on this conjecture. See [14, 17] for some recent developments.

### Conjecture 5.1

(Lovett [13]) Let $M\in \mathbb {R}^{n\times n}$ such that ${{\,\textrm{rank}\,}}(M)\le r$ and $M_{i,j}\ne 0$ for at most $\varepsilon n^2$ entries (*i*, *j*), where $\varepsilon \le 1/2$. Then there exist $X,Y\subset [n]$ such that $M_{x,y}=0$ for all $(x,y)\in X\times Y$ and $|X|=|Y|\ge n2^{-c\sqrt{\varepsilon r}}$ for some absolute constant $c>0$.
